# Continuing medical education challenges in chronic fatigue syndrome

**DOI:** 10.1186/1472-6920-9-70

**Published:** 2009-12-02

**Authors:** Dana J Brimmer, K Kimberly McCleary, Teresa A Lupton, Katherine M Faryna, William C Reeves

**Affiliations:** 1 Chronic Viral Diseases Branch, Coordinating Center for Infectious Diseases, Centers for Disease Control and Prevention, Atlanta, GA 30333, USA; 2 The CFIDS Association of America, Charlotte, NC 28222-0398, USA

## Abstract

**Background:**

Chronic fatigue syndrome (CFS) affects at least 4 million people in the United States, yet only 16% of people with CFS have received a diagnosis or medical care for their illness. Educating health care professionals about the diagnosis and management of CFS may help to reduce population morbidity associated with CFS.

**Methods:**

This report presents findings over a 5-year period from May 2000 to June 2006 during which we developed and implemented a health care professional educational program. The objective of the program was to distribute CFS continuing education materials to providers at professional conferences, offer online continuing education credits in different formats (e.g., print, video, and online), and evaluate the number of accreditation certificates awarded.

**Results:**

We found that smaller conference size (OR = 80.17; 95% CI 8.80, 730.25), CFS illness related target audiences (OR = 36.0; 95% CI 2.94, 436.34), and conferences in which CFS research was highlighted (OR = 4.15; 95% CI 1.16, 14.83) significantly contributed to higher dissemination levels, as measured by visit rates to the education booth. While print and online courses were equally requested for continuing education credit opportunities, the online course resulted in 84% of the overall award certificates, compared to 14% for the print course. This remained consistent across all provider occupations: physicians, nurses, physician assistants, and allied health professionals.

**Conclusion:**

These findings suggest that educational programs promoting materials at conferences may increase dissemination efforts by targeting audiences, examining conference characteristics, and promoting online continuing education forums.

## Background

Chronic fatigue syndrome (CFS) is a complex illness characterized by medically and psychiatrically unexplained fatigue that is not relieved by rest and is accompanied by symptoms of prolonged post-exertional malaise, unrefreshing sleep, impaired concentration and short-term memory, muscle and joint pain, headache, sore throat and tender lymph nodes [1,2]. The illness is clinically challenging because the etiology, pathophysiology and risk factors for CFS remain inchoate; there are no pathognomonic physical signs or diagnostic laboratory abnormalities; and, treatment is targeted at ameliorating symptoms rather than definitive cure.

In the United States, almost three percent of the adult population suffers from CFS [3]. While CFS was once described as an illness of educated upper income white women, population-based research shows that CFS affects all racial and ethnic groups and that persons of lower socioeconomic status are at increased risk [3-6]. Women are four times more likely than men to be affected [3,4]. More important, most people with CFS have been ill between 5 and 7 years; at least a quarter of them are unemployed or receiving disability because of the illness; and the average affected family forgoes $20,000 annually in lost earnings and wages (half of the median United States household income) [4,5,7,8].

In spite of the severe burden of morbidity that CFS imposes on the United States population, fewer than half those with the illness have consulted a physician and only 16% have been diagnosed and treated for CFS [5,6,8]. Lack of a timely CFS diagnosis delays intervention, which results in increased morbidity and work loss. Unfortunately, CFS is poorly understood within the medical community and many providers do not understand how to evaluate, diagnose and treat the illness [9]. Provider education specific to CFS affords a means of improving understanding of the illness, improving early diagnosis and treatment and reducing morbidity. One way of reaching health care providers is through formal continuing medical education programs.

The continuation of education for health care providers in the United States poses a challenge that has long been recognized by the medical community. Continuing medical education (CME) provides a means for practicing health care providers to maintain competence by keeping abreast of new knowledge in an increasingly dynamic profession [10], provides opportunities to learn new skills and incorporate new perspectives on disease management in clinical practice [11], and is required to meet requirements for re-licensure and recertification. Health care providers in multiple disciplines are required to participate in continuing education activities and obtain continuing education credits specific to their profession throughout the life of their career.

Examples of traditional CME activities include presentations at conferences, workshops and grand rounds, and these forums offer medical providers the opportunity to learn new skills and incorporate new perspectives on disease management in clinical practice [11]. There is not one preferred or most effective method of delivering continuing education instruction to enhance providers' performance [12-14]. In-person continuing education remains the preferred and most-used format but online learning is becoming increasingly popular [15]. While attending conferences is still a preferred format for physicians, randomized trials of Internet CME courses have shown that this medium is just as effective as traditional formats in increasing knowledge [16]. Self-study courses in print and electronic formats (e.g., audio CD or DVD) are also a common learning method.

The Centers for Disease Control and Prevention (CDC) through collaboration with the CFIDS Association of America developed a comprehensive strategy to educate primary and allied health care providers concerning evaluation, diagnosis, and management of patients with CFS. The Provider Education Project targeted primary care providers and allied health professionals through multiple interventions, some of which have been previously published [17]. One objective of the project promoted CFS continuing education to health care providers through professional outlets such as conferences, peer-reviewed journals, and health care resources on the Internet.

While CME is mandated for many healthcare professionals and much research has been conducted on the course format or learning methods, little is known about the outcome of dissemination of education at these conferences. To the authors' best knowledge and review of the literature, there are no data to suggest what types of conferences best result in increased dissemination. For example, does conference size impact distribution of materials? Does the type of conference facilitate more interaction? This paper reports the evaluation of 1) dissemination of CFS educational materials at provider conferences, and 2) the types of CME formats resulting in the greatest number of certifications.

## Methods

### Education materials at provider conferences

Conferences were selected by type of attendees. We targeted conferences with primary care providers (physicians, nurses, nurse practitioners, and physician assistants) and allied health professionals. These conferences included those sponsored by non-profit professional medical societies and national for-profit set conferences aimed at attracting primary care providers. At some of these conferences a CDC or CFS expert submitted a paper presentation, making these highly targeted conferences. We also selected conferences pertaining to CFS or specialized providers who may be more apt to treat chronic fatiguing illnesses. Last, conferences that targeted less represented medical groups were included on a periodic basis to identify emerging opportunities (i.e., medical societies for different gender/ethnic providers). The CFS provider education booth displayed the self-study courses and CFS provider education materials such as bibliographies, conference materials, and resource guide.

To evaluate effectiveness of distributing CME materials through provider education booths at conferences, we tracked booth attendance, compiled entries to reply devices at the booth, and tracked use of conference ID badges to request additional information. Ethical consent was not required as most conferences now use electronic tracking devices for outreach and promotional purposes. For each conference, staff kept records regarding CFS presentations the number of CME course requests and the number of training materials disseminated. At selected conferences promotional activities to direct conference attendees to the booth included pre-conference direct mailings to registered attendees, printed advertisements in conference packets or programs, web advertisements for selected health care sites, conference signage, and sponsorships.

Because booth attendance varies directly with the size of the overall conference, crude attendance counts do not permit standardized comparisons across multiple variables nor do such counts reflect interest by or penetration of the target population. Thus, our analysis involved booth attendance rate, calculated as the number of booth visits divided by the total number of conference attendees. The conference sponsor provided the overall conference attendance number. The average booth attendance rate for all conferences was 5%, and for one-quarter of the meetings at least 15% of all attendees visited the CFS continuing education booth. Thus we defined a 15% booth visit rate (the upper quartile) as successful conference exposure to continuing education materials.

We coded conference type into the following categories: *primary care *(e.g., family physicians, nurse practitioners or physician assistants), *allied professional *(e.g., physical or occupational therapists), *illness related *(e.g., CFS or fibromyalgia), and *special mixed population *(e.g., health educators, gender/ethnic medical organizations, or medical specialists). Information on the geographic scope of the conference was categorized as National (national membership and conference held in a major U.S. city), Regional (conferences attracting participants from particular geographic regions), and State (sponsored by a state medical societies or attracting attendances from one state).

Conference size was categorized as n < 1000 persons for small conferences, and n > 1000 for large conferences. CFS presentations at conferences were coded yes or no. We used both descriptive statistics and logistic regression analysis (SAS version 9.1, SAS Institute, Cary, North Carolina) to analyze conference data.

### CFS CME course

Experts in CFS from the CDC, academic institutions, clinical practice, and patient advocacy organizations convened to develop a continuing education self-study CFS course with the following objectives: 1) to outline the process for proper evaluation of persons suspected to have CFS; 2) to present a diagnostic algorithm for CFS, according to criteria of the 1994 International Case Definition; 3) to discuss management strategies for CFS; and 4) to improve providers' understanding as to the wide-ranging impact of CFS on patients' lives.

CDC served as sponsor organization for all continuing education accreditations: 1) the Accreditation Council for Continuing Medical Education for physician credits; 2) the American Nurses Credentialing Center's Commission on Accreditation for nursing, 3) and, the International Association for Continuing Education and Training for continuing education units. Certification was awarded for the following groups: 1) continuing medical education physicians (CME-P), 2) continuing medical education non-physician (mostly physician assistants and nurse practitioners) (CME-N), 3) continuing nursing education (CNE), 4) and, continuing education units (CEU) (other health care professionals such as occupational and physical therapists). Course participants were given two opportunities to score 70% or higher on a learning assessment and were required to complete a course evaluation.

Continuing education materials were promoted by paid advertisements at conferences, on websites, and in peer reviewed journals. The CME course was promoted at the CFS Provider Education booths through all mediums: print, DVD/video, and online. Print courses were distributed on site at conferences, DVD/video requests were taken on site or through a postcard, and the online course was advertised on handouts.

The self-study course was made available free of charge as printed text, a DVD or VHS video, and online. It was distributed at conferences and made available through advertisements in peer review journals. The print and online courses have indexed printed text and two case study reviews. The video presentations provide a fundamental overview of CFS, two case study reviews, and patient interviews. The materials were approved for continuing education credit, based on the average time for course completion and type of credit, as determined by course reviews performed by multidisciplinary professionals. The print and online course credits are based on a two-hour completion time, while the video course credit is based on a three-hour completion time. After completing the didactic part of the print or video module, health care providers submit a completed post-test and course evaluation by fax or postal mail and receive continuing education credits verified by a certificate of participation. Submission of information by web users occurs at the end of the online session, at which time they can print a certificate of participation. Providers registering for the online course were asked to voluntarily provide anonymous data on race, sex, age, and geographic region. As with all CME courses, no ethical approval is required as persons receive credit for course completion.

## Results

### Conferences - descriptive statistics

Between May 2000 and June 2006, the provider education project exhibited at 57 conferences, attended by 232,779 individuals and disseminated materials to over 11,000 persons (5% of attendees). Conference attendance size ranged from 75 to 12,000 with an average of 4,083. Audiences were diverse and included physicians, physician assistants, nurses, nurse practitioners, public health professionals, allied health professionals (psychologists, physical therapists, and occupational therapists), medical residents and students, disability attorneys, and medical specialists.

Seventy-seven percent of the conferences were National (n = 44), followed by 12% Regional (n = 7), and 11% State (n = 6). On average large conferences cost $5,490 while smaller and regional conferences cost approximately $1,750. Costs include exhibit related expenses, materials, travel, and conference fees. A CFS expert from CDC or an academic institution participated as a speaker or presented a poster at 22 of the conferences. Fourteen conferences (25%) met the 15% booth visit rate criteria and reached 2,140 providers (see Table 1).

**Table 1:** Conference booth visits

Conference Type	Geographic Scope	CFS Speaker	Conference Attendance (N)	Booth Visit Rate (%)	Promotion Activities
Primary care	National	No	2750	25	Yes
Illness related	National	Yes	600	33	No
Illness related	National	Yes	700	21	No
Illness related	National	Yes	330	15	No
Special population	National	No	650	19	No
Allied professional	National	Yes	75	53	No
Special population	National	Yes	350	21	No
Special population	National	Yes	500	24	No
Special population	National	No	550	18	No
Illness related	State	No	150	33	No
Primary care	State	Yes	500	40	Yes
Primary care	State	Yes	135	37	Yes
Special population	State	No	300	25	No
Special population	State	Yes	250	92	No

### Descriptive analysis

Further analysis of these 14 conferences showed that conference type, geographic scope, presentation of CFS research at the meeting, and magnitude of meeting attendance impacted booth visit rates.

The classification of conferences resulted in 8 *allied professional*, 5 *illness related*, 30 *primary care*, and 14 *special mixed population *(data not shown). Ten (71%) of the 14 conferences that met the 15% attendance criteria were classified as *illness related *(n = 4) and *special mixed population *(n = 6). State-level conferences had higher rates of booth visits implicating that geographic scope was a factor. Eighty-three percent of state level conferences met criteria for successful outreach compared to 20% of national conferences.

Conference size impacted interest at the CFS booth exhibit with smaller conference audiences attracting more booth visitors. Of the 57 conferences, 19 were small conferences, and 13 of the 14 conferences meeting the 15% criteria fell into this category (n < 1000). Of the 38 large conferences, only one met the booth visit criteria at 25%. When cross referencing geographic scope with conference size, 6 State (100%) level conferences fell into the small conference size category with only 1 Regional (14%) conference and 12 National (27%) conferences in this category.

The presence of a program about CFS delivered by a CFS expert or CFS researcher at a conference resulted in 9 of the 14 conferences meeting the 15% booth rate criteria and the overall highest booth visit rates, 92%, 53%, and 40%. Three of the 14 conferences had promotional activities.

Seventy-five percent (n = 43) conferences did not meet the booth rate visit criteria. Booth visits rates at these conferences ranged from 0.3% - 14% as compared to 15% - 92% for those conferences meeting cut-off criteria. Further analysis of the 43 conferences indicated that type of conference also impacted booth visit rates. For the non-successful conferences, 88% of both *primary care *and *allied professionals *types did not meet the criteria whereas only 20% of the *illness related *and 42% of the *special mixed population *did not meet the criteria.

Conference size was related to non-successful booth attendance; only 1 of the large size conferences met the 15% booth visit criteria. While the greatest number of booth visits were generated at large conferences (38 large sized conferences contributed a total of 9,655 booth visits compared to 19 smaller conferences with 1,793 total booth visits), the booth visit rates at large conferences was 4% compared to 19% of small conferences.

### Logistic regression

Table 2 presents results from the univariate logistic regression analysis. Conference type, conference size, and CFS presentation are significantly associated with successful conferences as measured by booth visits. For conference type, *illness related *conferences were more likely than *primary care *conferences to be successful (OR = 36.0; 95% CI 2.97, 436.34). Small conferences (OR = 80.17; 95% CI 8.80, 730.25) were more successful than large ones and a CFS presentation at the conference contributed to a greater likelihood of success (OR = 4.15; 95% CI 1.16, 14.83).

**Table 2:** Univariate logistic regression predicting successful conference booth visit rates

Conference type	β	SE	Odds Ratio	Wald Statistic
Intercept	-0.76	0.44		3.03
Allied professionals	-1.18	0.87	1.27	1.84
Illness related	2.15*	0.90	36.00	5.65
Special populations	0.47	0.58	6.75	0.66
Primary care	0		Reference	

**Conference geographic location**	**β**	**SE**	**Odds Ratio**	**Wald Statistic**

Intercept	-0.91	0.34		7.19
Regional or State	0.44	0.34	2.43	1.70
National	0		Reference	

**Conference size**	**β**	**SE**	**Odds Ratio**	**Wald Statistic**

Intercept	-1.42	0.56		6.34
Small	2.19**	0.56	80.17	15.17
Large	0		Reference	

**Conference presentation**	**β**	**SE**	**Odds Ratio**	**Wald Statistic**

Intercept	-1.08	0.32		11.07
Yes	0.71*	0.32	4.154	4.81
No	0		Reference	

* p < 0.5, ** p < 0.001

When all significant variables were entered into the model (conference type, conference size, and presentation), only conference size remained significant (OR = 48.39; 95% CI 3.88, 603.49) (see Table 3). However, this result is not surprising as chi-square tests show that conference size is significantly associated with conference type (23.0, p < 0.0001) and presentation (4.48, p < 0.05).

**Table 3:** Logistic regression predicting successful conference booth visit rates

Variable	β	SE	Odds Ratio	Wald Statistic
Intercept	-1.26	0.62		4.11
Conference type - allied	-0.27	1.28	1.16	0.04
Conference type - illness	0.68	1.04	3.03	0.43
Conference type - special	0.01	0.78	1.55	0.00
Conference type - primary	0		Reference	
Conference size - small	1.94*	0.64	48.39	9.08
Conference size - large	0		Reference	
Conference presentation yes	0.52	0.49	2.82	1.13
Conference presentation no	0		Reference	

* p < 0.5

### CME courses

Self-study course requests suggested that print and video materials from conferences yielded 3.8 times more requests as compared to requests stemming from advertisements in journals (see Table 4). For conferences, print requests were approximately 3 times greater than for the video materials. Print requests from journal advertisements were the most requested medium and greatly outnumbered any other types of requests stemming from journal advertisements.

**Table 4:** Course and material requests from conferences and journals

Continuing Education Self-study				Training Materials		
	**DVD/Video**	**Print**	**Total**	**Bibliography**	**Conference Materials**	**Resource Guide**

**Conference**	636	1721	2357	92	955	1253
**Journal**	42	568	610	19	14	67
**Total**	678	2289	2967	111	969	1320

Between January 2002 and June 2006, 4,686 providers requested print, video, and online self-study materials (Table 5). The online course accounted for 42% of the requests (n = 1,951), print for 41% (n = 1,927), and video for 17% (n = 808). About half (43%) of those who took the course online were awarded certificates. While print generated a large number of requests, only 7% of those who requested the print course submitted documentation to obtain credits. Video requests had both the lowest request and completion rates, only 2% of those who requested the video course were awarded certificates.

**Table 5:** Continuing education request and award by medium

Source	Course Requests N (%)	Course Award Certificates N (%)	Award Rate %
Online	1951 (42)	833 (84)	43
Print	1927 (41)	140 (14)	7
DVD/Video	808 (17)	18 (2)	2
Total	4686	991	21

Table 6 summarizes CFS education award certificates according to provider category. Allied health care professionals accounted for the most continuing education credits (CEU - 32%) followed closely by nurses (CNE - 31%), and physicians (CME - 25%). While non-physicians (CME-N) was low at 12%, this category comprised mostly of physician assistants and nurse practitioners is part of a large provider occupation base. Across all occupation categories the online course resulted in the most continuing education credit awards. The non-physician group had the highest print award numbers (n = 52) and the allied health group the highest online completions (n = 291).

**Table 6:** Continuing education awards by certificate type and medium

Source	CME-P N (%)	CME-N N (%)	CNE N (%)	CEU N (%)	TOTAL N (%)
Online	212 (84)	63 (52)	267 (88)	291 (92)	833 (84)
Print	33 (13)	52 (43)	35 (12)	20 (6)	140 (14)
DVD/Video	5 (2)	5 (4)	2 (4)	6 (10)	18 (2)
Total	250	120	304	317	991

CME-P - physicians

CME-N - non-physicians, physician assistants and nurse practitioners

CNE - nurses

CEU - allied health professionals, occupational and physical therapists

Table 7 presents demographic characteristics of persons who registered for the online CFS self-study course. The majority of registrants for the web-based self-study course were women (74%) and predominately white (85%). In terms of age, 36% fall into the 50-59 age category and 28% reported being age 40-49.

**Table 7:** Demographics for the web-based self-study course

Characteristic	N	%
Sex (n = 1751)		
Female	1296	74
Male	455	26
Age (n = 1437)		
20-29	78	5
30-39	275	19
40-49	405	28
50-59	521	36
60+	158	11
Race/Ethnicity (n = 1596)		
African American/Black	50	3
Latino/Hispanic	60	3
Asian	50	5
White	1436	85

## Discussion

The CFS provider education project disseminated CFS self-study course opportunities and resources at 57 conferences over a five-year period. At 14 of these conferences, the educational project met the 15% booth visit criteria for reaching conference attendees. Significant factors associated with successful booth attendance included conference type, conference size, and CFS presentations as part of the planned conference agenda.

At least 1 of each of the conference types, *primary care, illness related, specialty mixed, and allied professionals*, met successful booth visit criteria. However, *illness related *and *specialty mixed population *conference categories resulted in 10 conferences with booth visits at or above the 15% rate. *Illness related *conferences focused specifically on CFS or similar illnesses. One explanation for the interest in CFS among providers at these meetings is that they treat CFS patients in their practices, or CFS may be a specialty interest.

The dissemination project targeted primary care providers, yet only 3 of 30 primary care provider conferences met successful booth visit rate criteria. Moreover, *illness related *conferences were significantly more likely than *primary care *to result in successful booth visits. However, 24 of 30 *primary care *conference were large size conferences (n > 1000). The fact that many of the *primary care *conferences were large may have influenced the *primary care *category. Future educational initiatives in the area of chronic fatiguing illness should consider conference size when planning to target primary care providers at conferences.

Conference size was the best predictor of successful booth attendance. Small conference size (<1000) was more likely to result in higher dissemination levels than large conference size (> 1000), and this result was confirmed in both the descriptive and logistic regression analysis. One hypothesis is that small conferences are not so overwhelming for participants and give attendees more flexibility and time in visiting conference exhibit booths.

Nine of the 14 primary care provider conferences were National in scope and represented 20% of all National conferences. Yet, the State category (with 5 of the 14 conferences falling in this category) yielded a higher rate of 83% for all State conferences. While the results were not significant in the logistic analysis, it is noteworthy as many providers attend national conferences sponsored by their disciplines and may overlook state level conferences. While national conferences provide broad opportunities for partnerships and networking with academics, researchers and clinicians that are often not available at smaller conferences, our study descriptively observed that state level conferences cannot be overlooked as one way to disseminate health education materials. State conferences are less costly to attend, require less travel time and are of shorter duration, which minimizes time commitment for the participant.

Conferences in which there was an additional CFS component (e.g., speaker or highlighted research) resulted in 64% of conferences meeting the criteria suggesting that an expert presentation or research in which CFS was the focus may have prompted a higher number of booth visitors. The presence of an expert presentation was a significant factor in successful booth visits.

Requests for the CME self-study course through conferences were more than 3-fold as compared to advertisements in journals. The most requested medium for all self-study materials through conferences and journal advertisements was print-based matter (77%). While data show that requests (by medium) for print and online courses are equal (~40% each), the online course accounted for 84% of the overall awards compared to 14% for the print course. The online course requires registration, online learning and examination, and instant submission when the module is completed - all of which can be conducted in one sitting, or in multiple shorter online sessions, whichever the user prefers. Print courses place the burden on the user to complete the course and submission for awarded credit.

Allied health professionals and nurses led the award certifications and accounted for 63% of all certificates. Physicians accounted for one-fourth of the awards. All occupation groups had higher certificate awards for the online course compared to either the print or DVD/Video course. However, the non-physician group (mainly physician assistants and nurse practitioners) had a more narrow range between online and print course completion with awards of 52% (n = 63) and 43% (n = 52), and preferred both the print and online course material.

Anecdotal evidence supports the finding that nurses and allied health professionals completed the course in high numbers. These providers are often involved in the management of CFS patients and therefore may be more likely to seek additional information. Conversely, it is plausible for physicians to have lower rates given time constraints in clinical practice.

Several limitations to this study must be noted. First, data collection for the self-study course evaluation occurred at the descriptive measurement level. However, descriptive, observational studies offer important contributions to research and evaluation when it is not feasible to conduct an experimental design and resources are limited. Second, the 15% booth criteria rate was selected as a marker for successful educational dissemination in this study. To our knowledge the literature makes no reference as to the definition of success in material distribution. However, since the overall booth visit rate for all 57 conferences is 5%, we feel this criterion was valid as a cut-off point for this study. Finally, while advances in Internet medical education technology allow for greater efficiency and access to a bigger population, methods for online observational data collection are subject to similar limitations as in-person data collection. For example, while 1951 people registered for the online course, only 833 (43%) completed the course. More research is needed to explain why 57% of people who registered for the course did not complete it.

### Implications

Dissemination of provider education materials is a challenge for CFS and other illnesses such as autism, Lyme disease, and chronic interstitial cystitis, and many outreach programs rely on the traditional method of continuing education to reach and educate providers. Health education and continuing education programs wishing to distribute provider medical education materials for awareness and continuing education credits awards may find these results useful in guiding resource planning and outreach efforts.

Healthcare conferences are still a popular event among providers and offer an opportunity to disseminate education materials through educational booths. Results of this research show that educational outreach efforts could benefit from conducting a needs assessment of how to reach the primary audience and best utilize resources. This assessment would include looking at the conference type, conference size, and investing in expert speakers at conferences. The types of materials handed out at such conferences should also be examined in the context of the project objectives. While print materials were the most popular in this research, they yielded the half the award certificates as the online course. Educators need to develop methods for steering potential educational booth visitors to the Internet. Dissemination of CME self-study courses as conferences was more successful than advertising the same course in journals.

## Conclusion

This study offers new insight into the promotion of CFS continuing education materials. Our study showed that CFS medical education dissemination efforts resulted in higher booth rate visits at conferences when the project visited illness related conferences, small size conferences, and conferences with CFS presentations. Booth visits at all conferences resulted in high levels of distribution of print CME course materials as well as a provider diagnostic and management guide. Award of CFS continuing education credits was most effectively accomplished through an online course despite equal distribution of requests for print and online courses.

## Competing interests

The authors declare that they have no competing interests.

## Authors' contributions

DB drafted the manuscript, participated in study coordination, and performed the analysis. KM, TL, and KF participated in the study design and coordination. TL and KF performed data collection. WR conceived of the study, participated in study coordination and helped to draft the manuscript. All authors read and approved the final manuscript.

## Pre-publication history

The pre-publication history for this paper can be accessed here:

http://www.biomedcentral.com/1472-6920/9/70/prepub
